# Development of a Smartphone-Based Expert System for COVID-19 Risk Prediction at Early Stage

**DOI:** 10.3390/bioengineering9070281

**Published:** 2022-06-27

**Authors:** M. Raihan, Md. Mehedi Hassan, Towhid Hasan, Abdullah Al-Mamun Bulbul, Md. Kamrul Hasan, Md. Shahadat Hossain, Dipa Shuvo Roy, Md. Abdul Awal

**Affiliations:** 1 Department of Computer Science and Engineering, North Western University, Khulna 9100, Bangladesh; raihanbme@gmail.com (M.R.); mehedihassan@ieee.org (M.M.H.); towhidhasang1@gmail.com (T.H.); 2 Electronics and Communication Engineering Discipline, Khulna University, Khulna 9208, Bangladesh; bulbul@ku.ac.bd; 3 Department of Electrical & Electronic Engineering, Khulna University of Engineering & Technology, Khulna 9203, Bangladesh; kamruleeekuet@gmail.com; 4 Department of Quantitative Sciences, International University of Business Agriculture and Technology, Dhaka 1230, Bangladesh; shahadat_qs@iubat.edu; 5 Department of Information Science & Engineering, Visvesvaraya Technological University, Jnana Sangama, VTU Main Rd., Machhe, Belagavi 590018, India; shuvoroydipa@gmail.com

**Keywords:** adaptive synthetic sampling, Android or web-based user applications, COVID-19 prediction, feature selection methods, machine learning classifiers

## Abstract

COVID-19 has imposed many challenges and barriers on traditional healthcare systems due to the high risk of being infected by the coronavirus. Modern electronic devices like smartphones with information technology can play an essential role in handling the current pandemic by contributing to different telemedical services. This study has focused on determining the presence of this virus by employing smartphone technology, as it is available to a large number of people. A publicly available COVID-19 dataset consisting of 33 features has been utilized to develop the aimed model, which can be collected from an in-house facility. The chosen dataset has 2.82% positive and 97.18% negative samples, demonstrating a high imbalance of class populations. The Adaptive Synthetic (ADASYN) has been applied to overcome the class imbalance problem with imbalanced data. Ten optimal features are chosen from the given 33 features, employing two different feature selection algorithms, such as *K* Best and recursive feature elimination methods. Mainly, three classification schemes, Random Forest (RF), eXtreme Gradient Boosting (XGB), and Support Vector Machine (SVM), have been applied for the ablation studies, where the accuracy from the XGB, RF, and SVM classifiers achieved 97.91%, 97.81%, and 73.37%, respectively. As the XGB algorithm confers the best results, it has been implemented in designing the Android operating system base and web applications. By analyzing 10 users’ questionnaires, the developed expert system can predict the presence of COVID-19 in the human body of the primary suspect. The preprocessed data and codes are available on the GitHub repository.

## 1. Introduction

COVID-19 is a highly contagious disease caused by the recently discovered coronavirus 2, causing severe acute respiratory syndrome (SARS-CoV-2). It is highly infectious, with alarming characteristics, leading to the current worldwide pandemic [1]. The typical symptoms, such as fever, headache, shortness of breath, dry cough, and loss of smell, can vary from person to person; for instance, it sometimes does not raise any symptoms in some groups of people. It is noteworthy to mention that smokers and drug-addicted people are highly affected by this novel virus. However, unaware interaction with surrounding healthy people increases day by day, leading to the spread rate of such a disease. More than 176 million COVID-19 cases were reported up to June 2021, including 12.6 million active cases and 3.8 million deaths [2]. COVID-19 not only causes death but also imposes terrible effects on mental health [3] and human behavior [4]. However, isolation is one of the primary solutions for fighting against the spread of COVID-19.

Researchers from different fields are currently working to discover solutions for combating COVID-19 by developing a computer-aided detection (CAD) system. The authors of many articles have employed machine learning (ML) agents with different datasets to build such CAD systems. There are plenty of publicly available datasets, including both statistical data and chest radiographic images. However, careful strategies should be considered while exercising those datasets, as there is a possibility of building an overfitted trained model [5]. Aljameel et al. [6] developed an ML-based COVID-19 prediction method from 287 COVID-19 patients’ real-time data with 20 features from King Fahad University Hospital. An AUC of 0.99 was achieved with the RF classifier using Logistic Regression (LR), RF, and XGB on COVID-19 patient datasets. Arpaci et al. [7] proposed a 14-feature classification model for identifying COVID-19 patients using data from 114 subjects. They applied six different classifiers: BayesNet, LR, lazy-classifier, meta-classifier, regression model, and decision tree (J48), and obtained the best accuracy of 84.21% from the meta classifier (classification via regression). Recently, Debjit et al. [8] proposed a novel Harris hawks optimization-based eXtreme Gradient Boosting (HHOXGB) algorithm with an improved objective function and applied it to clinical COVID-19 data and obtained 92.23% accuracy. Wang et al. [9] proposed an automatic Deep Learning (DL)-based system by using CT images, collecting 4106 instances of lung cancer patients’ CT images and Epidermal Growth Factor Receptor (EGFR) gene sequence information. This study used EGFR datasets to determine if there was a link between abnormalities seen on a chest CT scan and micro-level lung functional deficits. However, they did not consider the intensive care unit and mild COVID-19 patients’ data and the intra-slice thickness of the CT scans. Kumar et al. [10] worked on chest X-ray images to classify pneumonia and could also be applied to detect COVID-19 patients by applying both ML and DL techniques. The ML techniques considered by the authors comprise numerous well-known classifiers such as XGB, LR, and K-Nearest Neighbors (KNN), and, as a representative of the DL type, they used ResNet-152. Brinati et al. [11] aimed to identify COVID-19 positive patients by analyzing blood test data using an RF classifier, an alternative to RT-PCR. In addition, they built a web-based application to evaluate COVID-19 through clinical support tools. The authors trained and validated their models using the nested cross-validation approach, utilizing an online dataset from the Zendo platform. Cabitza et al. [12] trained and evaluated their model using three different datasets, including 1624 patients’ data with 52% positive patients. They received the best accuracy (88%) from both the RF and SVM classifiers on the OSR dataset and the second-most accuracy (86%) from the KNN algorithm on the COVID-19-specific CBC dataset. Again, Elaziz et al. [13] developed an image-based COVID-19 prognostication model, where they used Fractional Multichannel Exponent Moments (FrMEMs) for feature extraction and Manta Ray Foraging Optimization (MRFO) for feature selection, and obtained an accuracy of 96.09%. However, the proposed approach aims to build a robust ML-based COVID-19 prognostication system to decrease its spread by providing a user-friendly, cheap, fast, and flexible in-house user application. Dhiman et al. [14] proposed that twin support vector machines can be applied in terms of different classification-related problems; for example, wind turbine gearbox anomalies and conditions can be predicted [15,16].

COVID-19 has spread to every country in the world, and the most common symptoms of this disease are the same. Developed and developing countries were affected at different rates depending on their economic and social status. The core contributions of this work are listed below:

1. Excluding redundant features from the utilized dataset by applying two different algorithms for ablation studies, such as *K* Best and Recursive Feature Elimination (RFE);
2. Optimizing the ML models’ hyperparameters using Bayesian optimization;
3. Performing comprehensive comparison in terms of different evaluation metrics among different ML models to select the best performing classifier for the aimed task;
4. Finding the association between the significant features with the Apriori algorithm;
5. Developing an intelligent application system in both smartphone and web interfaces to prognosticate COVID-19.

## 2. Analysis Procedure

**Figure 1:**
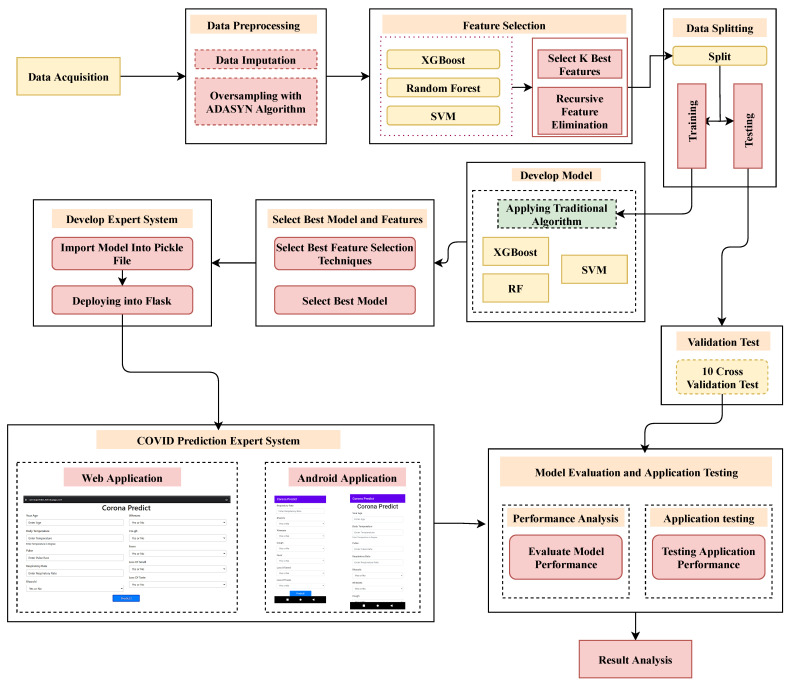
The recommended workflow for the identification of COVID-19 in conjunction with the proposed user application.

The workflow diagram of this aimed study is displayed in Figure 1, where the proposed method has been categorized into several steps.

Firstly, the dataset has been preprocessed for the subsequent steps in the proposed framework. It includes missing value imputation and re-balancing the dataset, followed by train-test splitting. Secondly, the feature selection method gives the essential and non-redundant attributes. Thirdly, XGB, RF, and SVM classifiers categorize the Covid and non-Covid patients. Finally, an expert system application was developed from the best-performing ML model for predicting COVID-19. The step-by-step methodology for conducting this study is described in greater detail in the following subsections.

### 2.1. Data Acquisition

**Figure 2:**
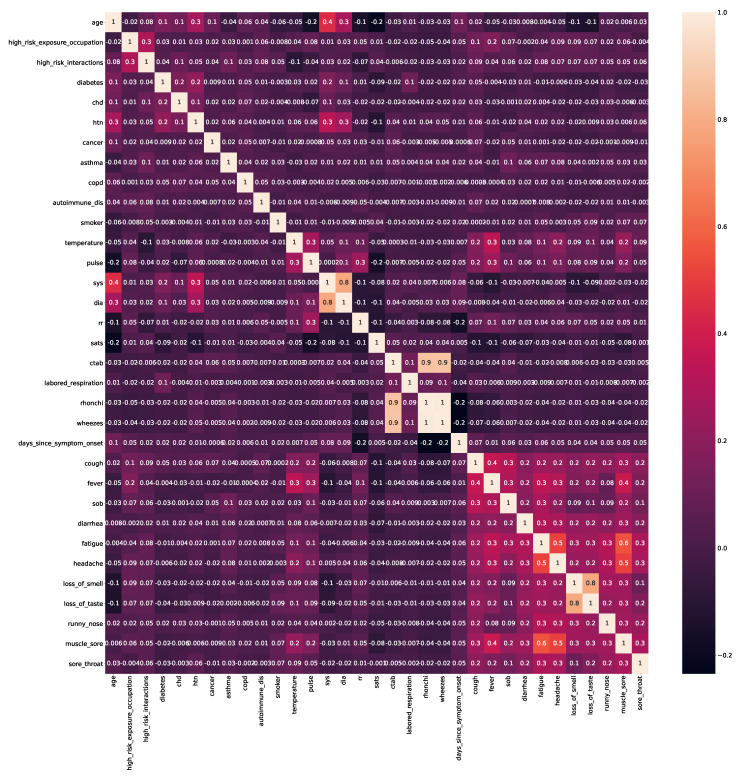
Heat–map representation of the features, dispensing the correlations between them.

In this study, a clinically useful COVID-19 dataset has been utilized [17], which has comprised Covid symptoms, for instance, ctab (Lungs Clear to Auscultation Bilaterally), loss of smell, labored respiration, diarrhea, rhonchi, wheezes, days_since_symptom_onset, fatigue, cough, fever, sob (Shortness of breath), muscle sore, headache, loss of taste, runny nose, and sore throat), epidemiological factors (for instance, age, high-risk Covid occupation, and high-risk interactions), comorbidities (for instance, diabetes, CHD (Coronary Heart Disease), HTN (Hypertension), cancer, asthma, COPD (Chronic Obstructive Pulmonary Diseases), autoimmune dis, and smoker), Vitals (for instance, temperature, pulse, Sys (Systolic), Dia (Diastolic), Rr (Respiratory Rate), and Sats (Oxygen Saturation). This dataset has 11,169 attributes, with positive and negative classes of 2.82% and 97.18%, respectively. The correlation of the features has been provided in Figure 2.

### 2.2. Preprocessing

In this article, the practical COVID-19 dataset is dealt with. It requires several preprocessing steps for imputing missing values and re-balancing the imbalanced data before engaging in the final ML-based classification. Moreover, such preprocessing will likely enhance the ML model’s performance [18]. However, the Multivariate Imputation has been applied by the Chained Equations (MICE) algorithm [19] to deal with the missing values. After that, the ADASYN algorithm has been used to eliminate the class imbalance nature of the dataset, where an adequate amount of artificial data has been created for the minority class. Finally, the feature selection method provides 21,578 instances with only 10 features.

The dataset used to carry out this research has 11,169 attributes, with 2.82% positive Covid results and 97.18% negative Covid test results for each sample. So, a significant class imbalance exists in the dataset, creating bias while classifying the target variables. This issue can be mitigated by generating a sufficient amount of synthetic data for the minority classes by oversampling these classes. There are two renowned techniques to oversample the minority classes: (i) the Synthetic Minority Oversampling Technique (SMOTE); (ii) the Adaptive Synthetic (ADASYN) sampling method, a generalization of the SMOTE algorithm. Both the algorithms mentioned above can create virtual data to solve the bias issue that occurred due to the class imbalance nature of the dataset. However, one disparity is that if there is any sample outlying in the minority class and that appears in the majority class, it will create a line bridge with the majority class. Another difference is that, while oversampling, the ADASYN takes the density distribution into account to accurately define the number of synthetic instances generated for the minority class, which is usually challenging to understand [20]. So, it is evident that the ADASYN algorithm assists in adjusting the decision boundary adaptively, relying on the problematic samples. Because of this exciting convenience, the ADASYN algorithm has been considered for solving the issue of the class imbalance nature of the dataset.

### 2.3. Classifiers

After the preprocessing, mainly three classification algorithms were applied: XGB, RF, and SVM. These classifiers provide improved results in many studies [21,22,23]. Furthermore, studies found significantly improved results by applying XGB in COVID-19 mortality prediction and prepared a clinically operable Covid decision support system using XGB for clinical staff [23,24]. This motivation leads to applying XGB along with other state-of-the-art RF and SVM. Therefore, XGB has mainly been described in detail with the important hyperparameters of RF and SVM.

**Figure 3:**
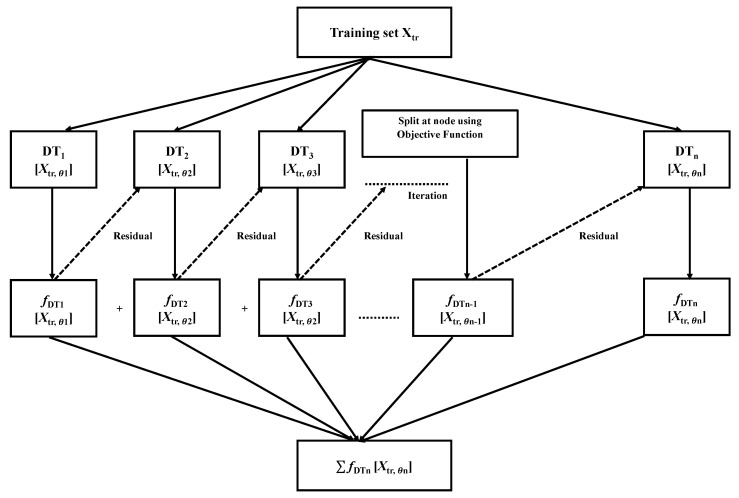
Flow chart of the working mechanism of the XGB algorithm in the XGB classifier. The highest accuracy was achieved by applying the XGB algorithm to the dataset.

XGB is a comprehensive ML system for tree boosting proposed by Chen and Guestrin [25], which was a winner of the Kaggle ML competition in 2015. Gradient Boosting is the base model of XGB, where multiple iterations co-occur. To optimize the specified loss function, the residual will correct the previous predictor at each iteration of gradient boosting, as illustrated in Figure 3. Regularization is applied to the loss function in order to establish the objective function in XGB, which is used to assess the model’s effectiveness [22,26].

Suppose there is a dataset with n samples, where $X_{a}$ is an independent variable and every variable has *m* number of features, therefore $X_{a}\epsilon R^{m}$. For every variable, there are corresponding dependent variables $y_{a}$, hence $y_{a}\epsilon R$. A tree ensemble model, such as $y_{a}$, predicts the dependent variable using the n additive functions of independent variables, as shown in Equation (1).

(1) $$y_{a}=\sum_{n=1}^{n}f_{DT_{n}\left(X_{a}\right)},f_{DT_{n}}\epsilon F,$$

where $f_{n}$ belongs to a leaf score independent tree structure, and *F* is the space of trees. After minimizing the above equation, it takes the following form in Equation (2) [25].

(2) $$L\left(\phi \right)=\sum l({\hat{y}}_{a},y_{a})+\sum \Omega (f_{DT_{n}}),$$

where the loss function is *l*, and Ω is the term that reduces the complexity of the model, which is defined in Equation (3).

(3) $$\Omega \left(f\right)=\gamma T+\frac{1}{2}\lambda {∥\omega_{i}∥}^{2}.$$

In the training phase, 10-fold cross-validation is employed to conduct experiments on main three ML classifiers. One fold is used as a testing set in the outer loop, and the remaining nine folds are utilized in the inner loop for the model’s training and hyperparameter optimization [18]. The Bayesian optimization algorithm has been employed to optimize the hyperparameters of all the classifiers [23]. Table 1 confers different important hyperparameters to be tuned using Bayesian Optimization. The intuition behind Bayesian optimization is illustrated in the following paragraphs.

To revolutionize the performance of the classifiers employed in the classification task, important hyperparameters must be optimized. In this study, mainly three classification algorithms, such as Random Forest (RF), Support Vector Machine (SVM), and eXtreme Gradient Boosting (XGBoost), have been utilized. Each classifier mentioned earlier has some distinct hyperparameters, which the Bayesian Optimization technique has tuned. For instance, Gamma (γ) and Cost (*C*) parameters have been tuned for SVM classifiers, where the Radial-Basis Function (RBF) kernel is controlled by the Gamma parameter. Furthermore, the TSVM with linear kernel and TSVM with RBF kernel has been employed in this research to compare these two variants, and the prevailing SVM [27,28]. While using the TSVM, the non-parallel hyperplanes are required to be controlled by tuning several hyperparameters, such as $c_{1}$, $c_{2}$, $e_{1}$, $e_{2}$, $v_{1}$, $v_{2}$. The broadness of the decision region depends on the lowness of the Gamma parameter, and the misclassification of the training instances is controlled by the Cost function. In the case of XGBoost, seven hyperparameters have been optimized: n_estimators define the total boosting stages; maximum depth of the estimator is denoted by max_depth, learning_rate controls the activities of each tree; Gamma represents the minimum loss; min_child_weight signifies the least summation of instance weight, colsample_by_tree represents the column subsample, and n_jobs symbolizes the parallel threads. In addition, the four hyperparameters for RF (criterion, max_depth, max_features, n_estimators ) have been optimized, where the criterion estimates the split quality and the maximum number of features is denoted using max_features.

The reason behind choosing Bayesian optimization is that it performs better than random search, grid search, or manual search techniques for tuning the hyperparameters of the classifiers. The most crucial advantage of Bayesian Optimization is that it keeps the previous evaluation in memory and helps in tuning the hyperparameter in a probabilistic manner. The important steps of Bayesian optimization are: (1) define a cost function; (2) search space; (3) number of iterations; and choose a search algorithm. Initially, there is a requirement to define a cost function; for instance, in this proposed approach, the 10-fold cross-validation loss has been considered in the cost function, followed by the task of searching space at the boundary of 0 and 1. In the last step, the number of iterations is fixed, and the best algorithm is chosen. In this proposed approach, the Tree-structured Parzen Estimator (TPE) algorithm has been considered by HyperOpt to finalize the optimization process.

### 2.4. User Application Design

The best-performing ML classifier from an experimental setting is deployed into a pickle file for developing a user-friendly application. Binary protocols are implemented in the pickle module so that a Python object structure can be serialized and deserialized into a bytes stream, and then the bytes stream can be converted into an object hierarchy again. However, in a real-world scenario, the practice of pickling and unpickling is widespread as it allows one to quickly transfer the data from one server/system to another and then store it in a file or database [29]. Then, flask, a Python web framework, is deployed to integrate the pickled model with the web application. Compared to the Django web framework, the Flask web application is more precise in everyday scenarios; hence, it is considered more Pythonic [23]. Besides, a user-friendly web application has been designed through which end users can easily input the data to test whether they are affected by COVID-19 or not in a probabilistic fashion. A higher probability indicates that the user has a higher risk of being COVID-19 affected and vice versa. High-risk users should follow proper guidelines and take medication for COVID-19 as early as possible.

## 3. Experimented Outcome and Discussions

This study comprised two types of analysis: (i) results related to classification model building; performance analysis of the model; and identification of essential features and the best classifier (see Section 3.1) and (ii) employment of the best classifier to develop an expert system discussed in Section 3.2.

### 3.1. The Outcome of ML Classifiers

In this study, 70.0% data was used to train the model and 30.0% to evaluate the trained model. The *K* Best and RFE algorithms have been practiced on the training dataset and also output 10 essential features (see Table 2).

1. Age;
2. Temperature;
3. Pulse;
4. Respiratory rate;
5. Bronchi;
6. Wheezes;
7. Cough;
8. Fever;
9. Loss of smell;
10. Loss of taste.

From Table 3, it is noteworthy that the Select *K* Best feature selection method provides better results than the RFE feature selection approach. The performance of the XGB classifier [30] has been significantly enhanced due to the Select *K* Best feature selection process. The XGB classifier demonstrates the highest metrics values compared to the other ML classifiers, such as RF and SVM (see Table 3). In the end, the XGB classifier, in conjunction with Select K=10 Best features, has provided the best result for the article.

**Figure 4:**
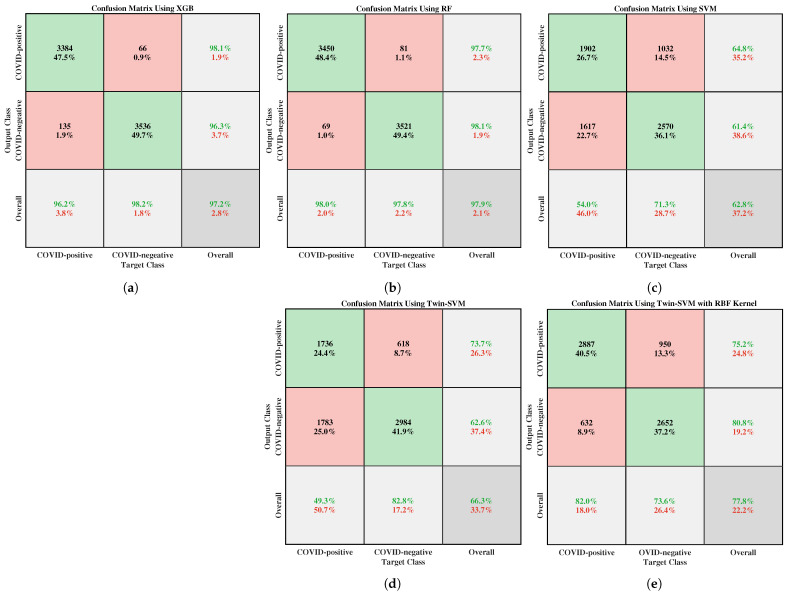
Confusion matrices of (**a**) XGB, (**b**) RF, (**c**) SVM, (**d**) Twin-SVM with linear kernel, and (**e**) Twin-SVM with RBF kernel for evaluating their class-wise performance.

Furthermore, the class-wise COVID-19 prognostication results are displayed in the confusion matrix, which is the visual representation of the classification performance. Figure 4a–c depict the confusion matrices for the XGB, RF, and SVM classifiers, respectively.

In addition, it has also been endeavored to test the results of the COVID-19 patient classification using the two well-known variants of Twin Support Vector Machine (TSVM) [27,31] because of its high-speed computational capacity. Other exciting advantages of using TSVM are that it ascertains two non-parallel hyperplanes instead of one hyperplane in the case of conventional SVM, and it can automatically unearth two-dimensional projections of the data. The perks of TSVM have been projected using both tabular and pictorial depictions. For instance, Table 4 delineates the comparative performance of traditional SVM and its two variants; Figure 4d,e visualize the confusion matrices of TSVM with linear kernel and TSVM with RBF kernel. From the tabular and graphical illustrations, it can be concretely decided that, although two variants of SVM perform better than the conventional SVM, the performance of XGB far outweighs the rest of the classifiers, including the TSVM. This is because the variants of TSVM have not been utilized for the further analysis of the proposed research work.

The first two diagonal cells of Figure 4 expose the corresponding number of trained networks and the percentage of proper classifications. For the best-performing XGB classifier, 66 of the COVID-19 cases are incorrectly classified as non-Covid, corresponding to 0.9% of all the 7121 examples in the data. Likewise, 135 of the non-Covid samples are falsely classified as COVID-19, corresponding to 1.9% of all the data. Furthermore, the XGB classifier has outputted a 98.1% positive predictive value, outperforming the RF and SVM classifiers, respectively, by the margins of 0.4% and 33.3%. Again, that comparative analysis of those three confusion matrices explains that the XGB classifier provides fewer false-positive and false-negative COVID-19 identification results with better true positive and negative while applying the K=10 Best feature selection.

**Figure 5:**
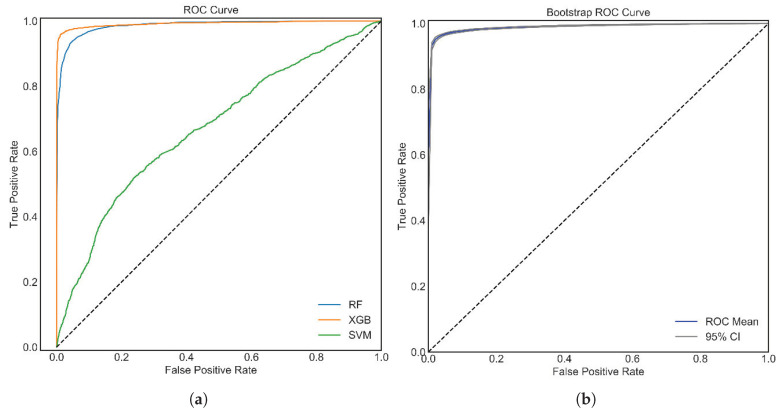
(**a**) ROC of RF, XGB, and SVM; and (**b**) bootstrap ROC analysis of XGB. The XGB classifier outperformed all the other algorithms.

Again, all the classifiers are further evaluated in terms of ROC curves and their AUC values. Figure 5a visualizes the ROC for the main three classifiers, which recognizes that the optimized XGB affords better results in terms of ROC compared to SVM and SVM. Higher AUC values offer robust performance in differentiating between the target classes. The experimental results reveal that the XGB classifier has the highest possible AUC of 98.0%.

The optimized XGB classifier has offered the best results in this study, as is evident from the above outcomes and discussions. The further assessment of the best performing XGB classifier has been accomplished using a bootstrap ROC, as attested in Figure 5b. In addition, the ROC 100 times (nboot=100) has been replicated, and thereby the corresponding results offer a 95% confidence interval.

#### 3.1.1. Cross-Validation, Analysis of Variance (ANOVA), and Multi-Comparison Test

The earlier results are based on the random train-test split, where 70.0% of the dataset was adopted in training and 30.0% of it in the testing phase. The 10-fold cross-validation (10-fold CV) was considered for additional robustness testing. This is done 10 times to estimate the model’s overall performance. In Table 5, the performances of the main three classifiers using a 10-fold CV are numerically presented. The mean accuracy for the XGB applying the 10-fold CV has the highest value of 96.68%. In contrast, the SVM offers 63.62%, and RF provides 90.77% cross-validation accuracy.

**Figure 6:**
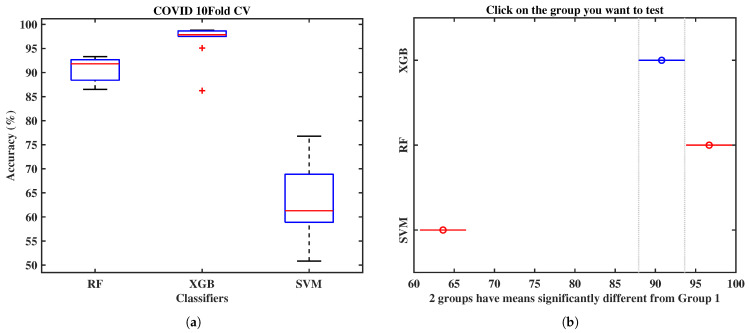
(**a**) Box and Whisker plot of 10-fold CV of the main three ML classifiers and (**b**) a multi-comparison test of the three trained models in this article.

The Box and Whisker plot in Figure 6a of those three models explains that the XGB classifier produces the best results, showing very few inter-fold variations and the highest mean accuracy.

The statistical significance of this study is carried out using the ANOVA test, employing a 10-fold CV. Additionally, the Multi-comparison Analysis (MCP) has been adopted, which has critical statistical properties. Failing to account for them leads to three induction algorithm pathologies: attribute selection errors, overfitting, and oversearching. However, a significant statistical analysis has been performed and calculated the *p*-value, $4.8\times 10^{-14}$ (p<0.001, statistical significance). This critical analysis has revealed statistically significant results. The optimized XGB result is statistically more important than the RF and SVM manifested in Figure 6b, as is apparent from the multi-comparison test.

**Figure 7:**
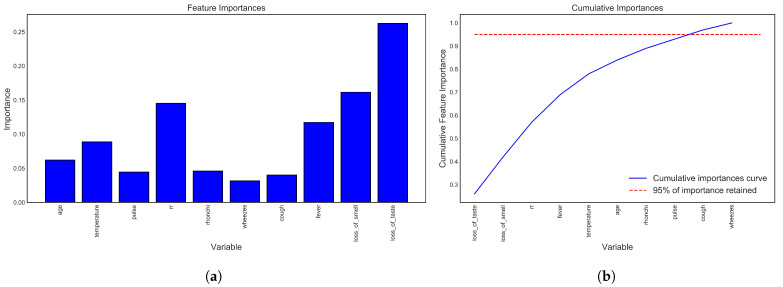
Feature importance and cumulative importance using XGB. The most important feature is loss_of_test, which is a noteworthy discovery from the dataset investigation. Then, (**a**) showed the selected 10 critical features, and (**b**) visualizes the cumulative feature importance curve, which also indicates that it grows to 95% above.

The importance of the t10 selected best features is exhibited in Figure 7a using the best performing XGB classifier. The most important features are loss of taste and smell, and the least essential feature among the 10 is wheezes. Cumulative importance (CI) calculates the most important features and describes how the second-most important features improve the performance. Performing CI, the 10 features are shown in Figure 7b, and the performance is almost 95%.

**Figure 8:**
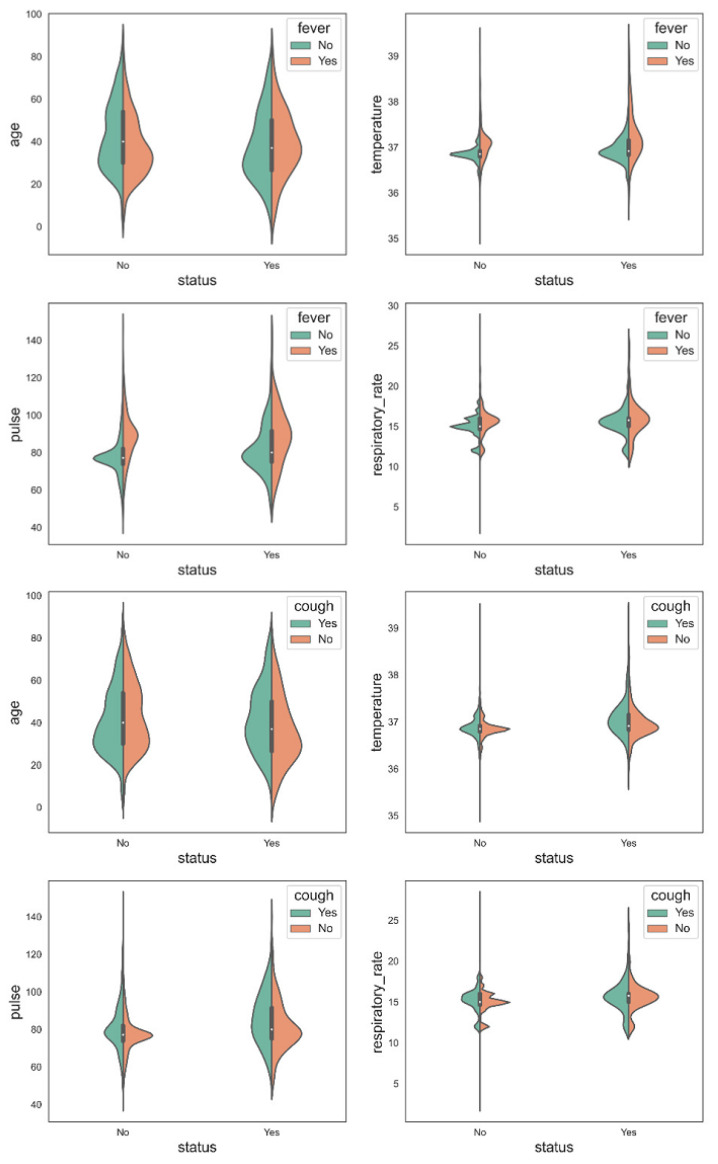
Violin plot of data distribution in terms of fever and cough.

The violin plot displayed in Figure 8 is also created to visualize the data and data distribution in terms of fever and cough. It clearly distinguishes between Covid and non-Covid patients [32]. Therefore, an optimized XGB model using 10 important features (age, temperature, pulse, respiratory rate, bronchi, wheezes, cough, fever, loss of smell, loss of taste) has been used to design an intelligent app that is discussed in Section 3.2 [33].

Furthermore, from Table 6, it is evident that the model performance has been bestowed on the selected features. Without feature selection, the performance of RF and XGB is almost similar, with an accuracy of 97.89% and 97.18% separately. However, the Matthews correlation coefficient is 95.79% and 94.37% for RF and XGB, respectively. The results using the 10 best features are slightly better than this. So, it can be concluded that using the 10 features resulting from SelectkBest in collaboration with the optimized XGB provides the best results. From these results, the violin plots have also been carried out (see Figure 8).

**Figure 9:**
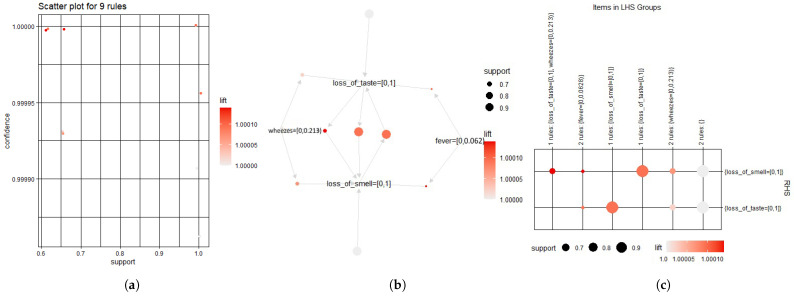
Association rules (**a**) scatter plot, (**b**) support and lift, and (**c**) items in the left hand side (LHS) and the right hand side (RHS) group.

The most important rules are derived from a set of 10 attributes. It has been attempted to discover a relationship between the selected features due to this result. These nine rules are extracted based on confidence (35%) and support (95%), and Figure 9 depicts the relationship between those variables and the rules for Covid patients.

#### 3.1.2. State-of-the-Art Comparison

The previous discussion reveals that 10 features have been selected by employing Select-*K* Best feature selection and are shown to outperform using XGB. The results are also comparable with other contemporary studies. In Table 7, Awal et al. [23] worked on the same dataset where the feature number and total instances were 33 and 21578. They applied mainly three different ML algorithms and got the best accuracy of 98.63% from XGB with 33 features, but no expert system was built there. On the other hand, with 10 significant features, the expert system developed using the proposed approach achieved 97.91% accuracy in the XGB model, which is quite close to Awal et al. [23] work. Kumar et al. [10] also developed their prediction model by applying RF and XGB. Arpaci et al. [7] achieved the best accuracy of 97.7% by applying XGB on the dataset containing three features and 5840 instances. Debjit et al. [8] used a dataset consisting of 20 clinical features and obtained 92.54% accuracy. The proposed system was based on 10 features, and the cases were 21,578. However, it is obvious that the better accuracy is 97.91% from XGB, 97.81% accuracy from RF, and 73.37% is obtained from SVM.

### 3.2. Mobile and Web Application Development

**Figure 10:**
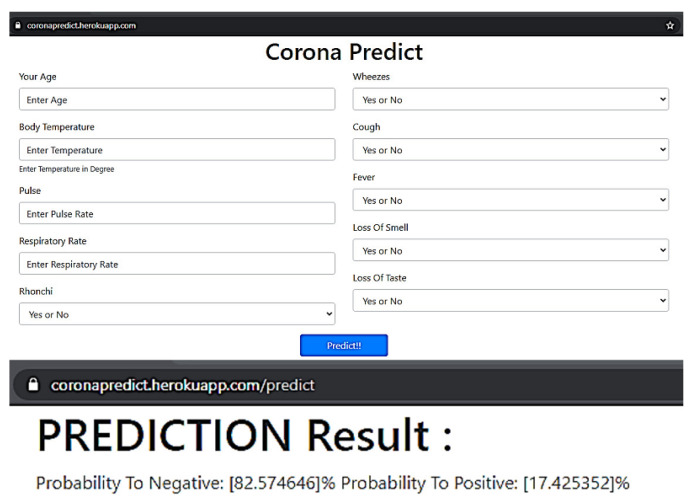
Screenshot of the designed web application for COVID-19 prognostication, deploying the proposed framework.

**Figure 11:**
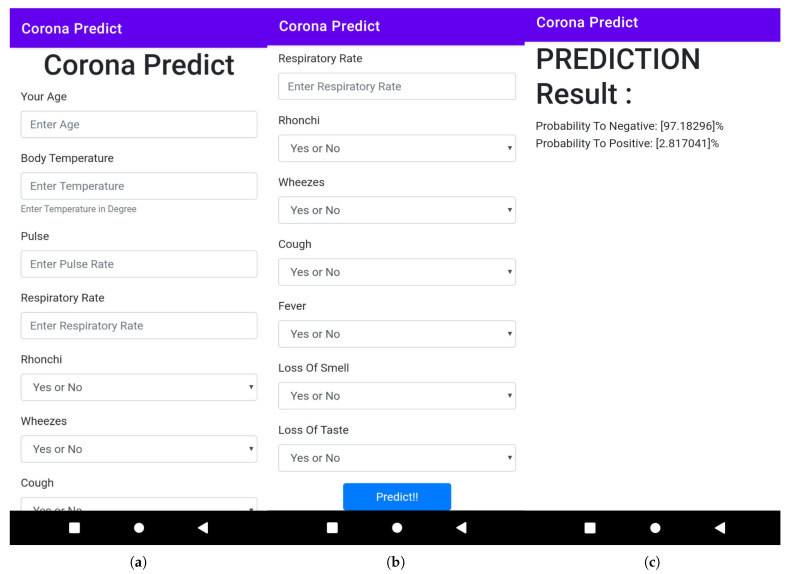
Several screenshots, such as (**a**) queries from users, (**b**) queries from users, and (**c**) predicted results of the implemented mobile application for COVID-19 prognostication, deploying the proposed framework.

A user-friendly web and mobile application have been developed from the model on which end users can easily input the data to test whether they can affect COVID-19 in a probabilistic fashion or not. Here, end-users input 10 data points such as age, body temperature, pulse rate, respiratory rate, bronchi, wheezes, cough, fever, loss of smell, and taste. The higher probability indicates a higher risk of affective COVID-19 and vice versa. The high-risk user should follow proper guidelines and medication against COVID-19. The web application, as well as the mobile interface of the proposed framework, have been presented in Figure 10 and Figure 11, respectively.

After input, those 10 data that predict the button return the following Figure 11 with the positive and negative probability percentages. Here is the screenshot of the newly designed smartphone application. End users can here input these 10 data and predict the probability of positive or negative.

## 4. Conclusions

COVID-19 has altered global, social, and economic conditions. The sturdiness of international relations has been tested, whether on a unilateral or multilateral basis. The most obvious consequences of this disease are economic recession, a global governance crisis, trade protectionism, and increased isolationist sentiment. There have been restrictions on international exchanges of people, culture, and travel. Nevertheless, this is only the beginning. The world will be able to maintain stability in the future when faced with similar challenges because people will overcome the pandemic. If COVID-19 is detected early, it may be possible to defeat the virus. Furthermore, here, a model is built for the early detection of COVID-19 to facilitate end-users by quickly testing whether they are affected by COVID-19 in a probabilistic fashion. A higher probability indicates that the user is more likely to be affected by COVID-19 and vice versa. Mainly, three ML algorithms have been utilized, namely XGB, RF, and SVM, to establish this model, which obtained an average accuracy of 97.91% for XGB, 97.81% for RF, and 73.37% for SVM. Finally, an expert intelligence system has been developed using the outcomes of this study, and it is aimed to be highly efficient and accurate in predicting and classifying COVID-19, as its symptoms are the same. This application can be applied to any country’s people. In the future, deep learning and transfer learning may be integrated into the proposed algorithm to make the application more efficient. The sample size of the dataset can be enlarged to make the analysis more feasible, accurate, and valuable. The current dataset has been stored in the GitHub repository (https://github.com/awalece04ku/Covid-ML/) (accessed on 26 February 2022) for future app usability testing and performance measurement.

## Figures and Tables

**Table 1:** Hyperparameters of various ML-based classifiers to be tuned.

Hyperparameters	Short Description
	**XGB classifier**
Estimator numbers	Multiple boosting rounds worth of gradients were used to boost these trees
Learning rate	How much the contribution of each tree will shrink
Number of jobs	The number of XGB classifier parallel threads used
Maximum depth	The tree’s node count (the best value depends on the interaction of the input variables)
Gamma values	The bare minimum loss reduction necessary to allow for further leaf node partitioning
Minimum child’s weight	The bare minimum in terms of child instance weight
Column sample numbers	The ratio of columns’ sub-samples during tree construction
	**SVM classifier**
Cost (C)	A regularization parameter that controls the trade-off between classification accuracy on the training instances and margin maximization
Gamma (γ)	A kernel parameter of the radial basis function that defines the reciprocal of standard deviation. It describes how the inverse of the radius influences support vector data points
	**RF classifier**
Estimator numbers	Represents the number of decision trees in the forest
Maximum depth	Denotes the decision tree’s maximum depth
Minimum samples’ split	Minimum instances needed to split a tree’s internal nodes are indicated by this value
Minimum samples’ leaf	Minimum number of instances required to be at leaf node is denoted by this value
	**Twin SVM classifier**
$c_{1}$ and $c_{2}$	Represent the penalty parameters that control the classification accuracy and margin maximization
$e_{1}$ and $e_{2}$	Denote the column vectors of ones of appropriate dimensions depending on the dataset
$v_{1}$ and $v_{2}$	Symbolize the parameters that ascertain the tradeoff between the support vector and the interval error

**Table 2:** The decided attributes come from two different selection algorithms. Various features have been chosen to be applied via RFE. By applying *K* Best, the last 10 features are selected for applying classification models.

Recursive Feature Elimination (RFE)			*K* Best
XGB	RF	SVM	
high_risk_exposure_occupation	age	diabetes	age
htn	high_risk_exposure_occupation	copd	temperature
smoker	high_risk_interactions	temperature	pulse
cough	temperature	ctab	rr
fever	sys	wheezes	rhonchi
sob	rr	fever	wheezes
fatigue	sats	diarrhea	cough
headache	cough	fatigue	fever
loss_of_smell	fever	loss_of_smell	loss_of_smell
loss_of_taste	loss_of_smell	loss_of_taste	loss_of_taste

**Table 3:** Classification performance of different classifiers using RFE, select *K* Best, and without feature selection. The model’s performances have been proven by comparing several metrics like accuracy, F1_score, Kappa, and more. On *K* Best feature, best model performances (Accuracy, Precision, Sensitivity, Specificity) accomplished by XGB.

Different Performance Metrics (%)	Feature Selection Methods						Without Feature Selection Methods		
	*K* Best			RFE					
	RF	XGB	SVM	RF	XGB	SVM	RF	XGB	SVM
Accuracy (ACC)	94.38	**97.18**	63.91	95.82	66.53	62.12	97.88	97.18	62.80
F1_Score	94.43	**97.23**	66.77	95.88	71.88	71.31	97.91	97.24	65.99
False Positive Rate (FPR)	5.43	**3.75**	44.05	4.37	51.94	69.56	2.30	3.84	45.95
Kappa Statistic (Kappa)	88.76	**94.35**	27.68	91.65	32.78	23.69	95.76	94.35	25.45
Matthews Correlation Coefficient (MCC)	88.77	**94.37**	28.00	91.65	35.12	30.26	95.76	94.37	25.80
Precision (PPV)	94.67	**96.40**	62.48	95.73	62.50	57.80	97.76	96.32	61.38
Sensitivity (SEN)	94.20	**98.08**	71.68	96.02	84.59	93.08	98.06	98.17	71.35
Specificity (SPE)	94.57	**96.25**	55.95	95.62	48.05	30.43	97.70	96.16	54.05

**Table 4:** Comparative performance of SVM and its two variants.

SVM and It Variants	Performance Metrics (%)								
	ACC	Error	F1_Score	FPR	Kappa	MCC	PPV	SEN	SPE
SVM	63.91	36.09	66.77	44.05	27.68	28.00	62.48	71.68	55.95
Twin SVM with linear kernel	66.28	33.72	71.31	50.67	32.30	34.20	62.60	82.84	49.33
Twin SVM with RBF kernel	77.78	22.22	77.03	17.96	55.61	55.83	80.76	73.63	82.04

**Table 5:** A 10-fold cross-validation accuracy for the main three classifiers (RF, XGB, and SVM). The best Mean ± Std value is archived from XGB, which is 96.68±3.82.

Different Folds	RF	XGB	SVM
Fold 1	86.52	86.24	50.83
Fold 2	91.38	97.68	66.50
Fold 3	92.26	97.68	72.75
Fold 4	87.21	95.09	61.68
Fold 5	88.42	97.50	60.89
Fold 6	93.05	98.66	68.86
Fold 7	92.68	98.01	76.78
Fold 8	92.59	98.75	58.39
Fold 9	90.36	98.79	58.88
Fold 10	93.32	98.42	60.59
**Mean ± Std**	90.78±2.53	96.68±3.82	63.62±7.64

**Table 6:** Selected feature models performances using RF, XGB, and SVM are quantified in terms of accuracy, error, F1_Score, false-positive rate, and Kappa. The highest degree of precision, accuracy, and specificity is achieved via the efforts of RF.

Classifiers	Performance Metrics (%)								
	ACC	Error	F1_Score	FPR	Kappa	MCC	PPV	SEN	SPE
RF	97.89	2.11	97.91	1.96	95.79	95.79	98.08	97.75	98.04
XGB	97.18	2.82	97.24	3.84	94.35	94.37	96.32	98.17	96.16
SVM	62.80	37.20	65.99	45.95	25.45	25.80	61.38	71.35	54.05

**Table 7:** Comparison of accuracy between the existing outcomes and the proposed system.

Different Studies (year)	Feature Numbers	Sample Numbers	Algorithms	Accuracy (%)
Awal et al. [23] (2021)	33	21,578	RF	98.54
			XGB	98.63
			SVM	96.75
Wang et al. [9] (2020)	15	279	RF	80.00
			SVM	80.00
Kumar et al. [10] (2020)	69	1925	RF	93.00
			SVM	91.00
Arpaci et al. [7] (2020)	3	5840	RF	97.30
			XGB	97.70
Debjit et al. [8] (2022)	20	48,676	XGB	92.54
			RF	91.53
			SVM	84.54
**Proposed system (2022)**	**10**	**21,578**	**XGB**	**97.91**
			**RF**	**97.81**
			**SVM**	**73.37**

## Data Availability

The processed data, trained model, and codes related to this study are available at: https://github.com/awalece04ku/Covid-ML/ (accessed on 26 February 2022).
